# Deletion of *OSBPL2* in auditory cells increases cholesterol biosynthesis and drives reactive oxygen species production by inhibiting AMPK activity

**DOI:** 10.1038/s41419-019-1858-9

**Published:** 2019-08-19

**Authors:** Hongshun Wang, Changsong Lin, Jun Yao, Hairong Shi, Cui Zhang, Qinjun Wei, Yajie Lu, Zhibin Chen, Guangqian Xing, Xin Cao

**Affiliations:** 1 Department of Medical Genetics, School of Basic Medical Science, Nanjing Medical University, Nanjing, China; 2 Jiangsu Key Laboratory of Xenotransplantation, Nanjing Medical University, Nanjing, China; 3 The Laboratory Center for Basic Medical Sciences, Nanjing Medical University, Nanjing, China; 4 Department of Otolaryngology, The First Affiliated Hospital of Nanjing Medical University, Nanjing, China

**Keywords:** Cell signalling, Cell signalling, Clinical genetics, Clinical genetics

## Abstract

Oxysterol-binding protein like 2 (*OSBPL2*) was identified as a novel causal gene for autosomal dominant nonsyndromic hearing loss. However, the pathogenesis of *OSBPL2* deficits in ADNSHL was still unclear. The function of OSBPL2 as a lipid-sensing regulator in multiple cellular processes suggested that OSBPL2 might play an important role in the regulation of cholesterol-homeostasis, which was essential for inner ear. In this study the potential roles of OSBPL2 in cholesterol biosynthesis and ROS production were investigated in *Osbpl2*-KO OC1 cells and *osbpl2b*-KO zebrafish. RNA-seq-based analysis suggested that OSBPL2 was implicated in cholesterol biosynthesis and AMPK signaling pathway. Furthermore, *Osbpl2/osbpl2b*-KO resulted in a reduction of AMPK activity and up-regulation of *Srebp2/srebp2, Hmgcr/hmgcr* and *Hmgcs1/hmgcs1*, key genes in the sterol biosynthetic pathway and associated with AMPK signaling. In addition, OSBPL2 was also found to interact with ATIC, key activator of AMPK. The levels of total cholesterol and ROS in OC1 cells or zebrafish inner ear were both increased in *Osbpl2/osbpl2b*-KO mutants and the mitochondrial damage was detected in *Osbpl2*-KO OC1 cells. This study uncovered the regulatory roles of *OSBPL2* in cellular cholesterol biosynthesis and ROS production. These founds might contribute to the deep understanding of the pathogenesis of OSBPL2 mutation in ADNSHL.

## Introduction

Cholesterol is an essential constituent of organelle membranes in eukaryotic cells, where it is implicated in various cellular processes, including the regulation of membrane permeability, membrane trafficking, signal transduction, and endocytosis^1–5^. The intracellular cholesterol synthesis, efflux (egress), and uptake (ingress) are tightly controlled by the feedback on direct interactions of cholesterol or oxysterols with regulatory factors, such as 3-hydroxy-3-methylglutaryl-coenzyme A reductase (HMGCR), sterol responsive element-binding protein (SREBP) cleavage activating protein (SCAP), acyl-coenzyme A: cholesterol acyltransferase (ACAT) and liver X receptors (LXRs). These factors act as sterol sensors and effectively regulate cholesterol-homeostasis by a serial of post-transcriptional and post-translational mechanisms^6–9^. Increasing evidences suggest that deregulation of cholesterol-homeostasis is the causative or characteristic factor of numerous pathologies, including cardiovascular disease, cerebrovascular disease, metabolic diseases, tumourigenesis and neurodegenerative diseases^10–13^. It was also noteworthy that sensorineural hearing loss (SNHL) is observed as one of the characteristic features in atherosclerosis and some genetic syndromes that affect intracellular cholesterol synthesis or transport^14,15^.

Oxysterol-binding protein like 2 (OSBPL2, OMIM: 606731), a member of the oxysterol-binding proteins (OSBP) related proteins (ORPs) family, is known as a sterol sensor and transporter that modulates lipid/cholesterol metabolism, steroid hormone synthesis, cell signaling, vesicular trafficking and cytoskeleton formation^16–20^. Our previous work described the association of human mutations in *OSBPL2* with autosomal dominant non-syndromic hearing loss (ADNSHL) in a large affected Chinese family^21^, which was also reported in an affected German family by Thoenes et al^22^. The pathogenesis of *OSBPL2* deficits in ADNSHL was still unclear, however, the importance of cholesterol-homeostasis in inner ear as well as the unique sensitivity of the auditory organ to changes in cholesterol localization^23,24^ suggested that the potential role of *OSBPL2* in lipid/cholesterol-homeostasis was essential to the auditory function. In fact, the cellular function of *OSBPL2* has been reported to be associated with cholesterol and triacylglycerol homeostasis in distinct cell lines^25,26^. Escajadillo et al. found that reduced amounts of multiple steroid metabolites were observed in *OSBPL2*-knockdown H295R adrenocortical cells, and *OSBPL2*-silencing led to the suppression of key factors for cortisol production, resulting in increased cellular cholesterol and reduced 22(R)-hydroxycholesterol [22-(R)-OHC] and 7-ketocholesterol (7-KC)^16^. It was suggested that the *OSBPL2* deficits might result in the loss of tightly-regulated cholesterol-homeostasis response in inner ear and lead to the auditory dysfunction. Considering the ubiquitous expression of *OSBPL2* in diverse cell types and multiple cellular functions of this sterol sensor, the potential pathogenesis of *OSBPL2* deficits in ADNSHL still needed to be further investigated in the functional or dysfunctional context of specific cell types in vitro and in vivo.

In this study, OC1 auditory cell and zebrafish as well as their *Osbpl2/osbpl2b*-knockout (KO) mutants were used to investigate the potential role of *OSBPL2* in the regulation of cholesterol-homeostasis. Our findings showed that *Osbpl2/osbpl2b*-KO led to excessive cholesterol biosynthesis by inhibiting AMPK signaling pathway and induced the production of intracellular reactive oxygen species (ROS), which might be the causative factor of mitochondrial damage in *Osbpl2*-KO OC1 cells. This work would contribute to deep understanding of the molecular function of *OSBPL2* in auditory cells/inner ear, and provide an insight of the pathogenesis of *OSBPL2*-deficit implicated in ADNSHL.

## Results

### *Osbpl2*-KO interferes with AMPK signaling

**Fig. 1:**
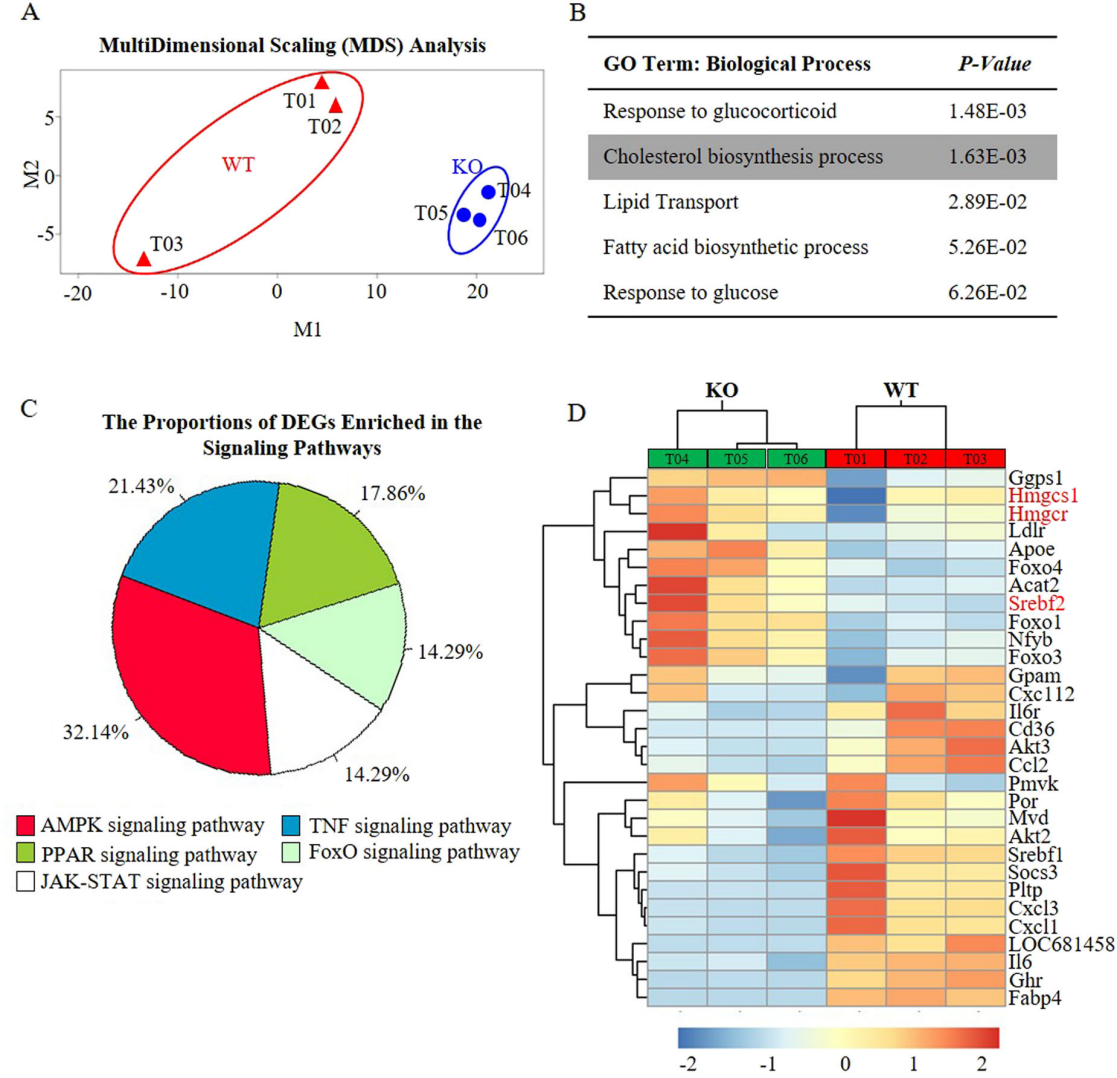
RNA-seq of *Osbpl2*-KO and wild-type (WT) OC1 cells. **a** Multi-Dimensional scaling (MDS) analysis of RNA-seq profiles collected from *Osbpl2*-KO and WT OC1 cells (*n* = 3). **b** The enrichment of biological processes was determined by gene ontology (GO) analysis. *P-value* was assessed by Benjamini procedure. **c** The gene enrichment was determined by KEGG pathway analysis, the % scale represented the proportions of DEGs enriched in the signaling pathways to total DEGs associated with cholesterol biosynthesis. **d** Genes enriched in AMPK signaling pathway were shown in the heat map. Data was normalized by logarithm of 2

RNA-seq was performed to identify genes and pathways affected by OSBPL2-function/dysfunction. The expression profiles were examined by using multidimensional scaling (MDS) analysis to visualize the profiles and by comparing differentially expressed genes (DEGs). MDS analysis showed that significant transcriptional changes were detected in *Osbpl2*-KO OC1 cells, with 1435 (11.66%) DEGs out of 12309 detectable genes (Fig. 1a and Fig. S1). GO analysis of this gene set revealed that the enriched clusters were related to the cholesterol biosynthesis, lipid transport and sterol metabolic process (Fig. 1b). Then, we analysed DEGs associated with cholesterol biosynthesis by KEGG pathway analysis. The results showed that AMPK signaling was predicted to be the most significant pathway (Fig. 1c). To validate this, we measured the expression of top-ranked genes in each cluster and additional genes involved in AMPK signaling pathway and performed hierarchical Euclidean distance analysis (Fig. 1d). In *Osbpl2*-KO cells, the majority of genes related to cholesterol biosynthesis were upregulated and clustered. These results suggested that *Osbpl2* was involved in the regulation of cholesterol biosynthesis and AMPK signaling pathway.

### *Osbpl2*/*osbpl2b* -KO inhibits AMPK activity and increases cholesterol synthesis

**Fig. 2:**
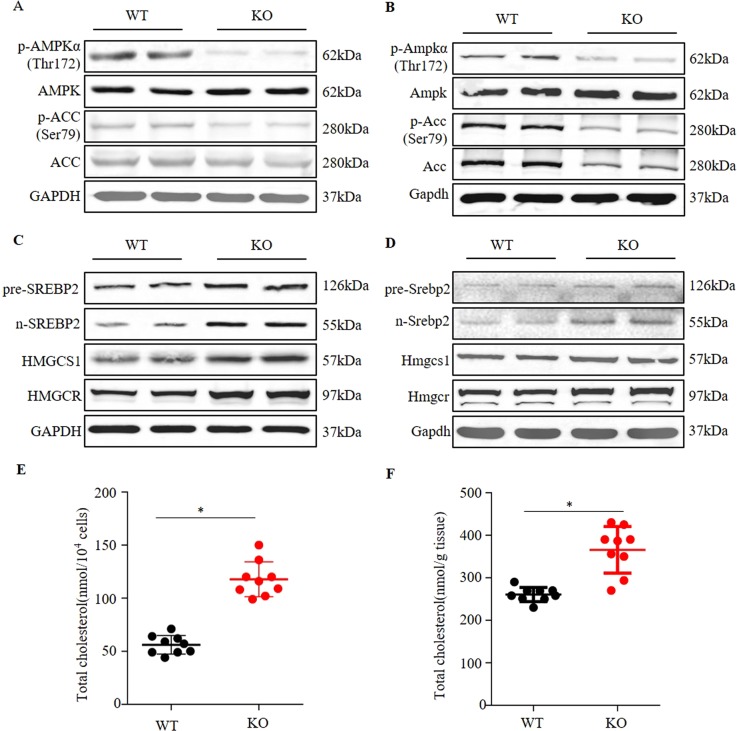
*Osbpl2/osbpl2b*-KO inhibited AMPK activity and increased intracellular cholesterol levels. **a** Western blot analysis of AMPK (total and Thr172), ACC (total and Ser79) and GAPDH in *Osbpl2*-KO/WT OC1 cells. **b** Western blot analysis of Ampk (total and Thr172), Acc (total and Ser79) and Gapdh in *osbpl2b*-KO/WT zebrafish inner ear. **c** Western blot analysis of pre-SREBP2, n-SREBP2, HMGCR, HMGCS1 and GAPDH in *Osbpl2*-KO/WT OC1 cells. **d** Western blot analysis of pre-Srebp2, n-Srebp2, Hmgcr, Hmgcs1, and Gapdh in *osbpl2b*-KO/WT zebrafish inner ear. **e** Total cholesterol levels in *Osbpl2*-KO/WT OC1 cells (*n* = 9), *p *<* 0.05. **f** Total cholesterol levels in *Osbpl2*-KO/WT zebrafish inner ear (*n* = 9), *p < 0.05

To investigate the effect of *Osbpl2*-KO on AMPK signaling pathway and cholesterol synthesis, the protein levels of phosphorylated AMPK-Thr172 and total AMPK were measured in *Osbpl2*-KO/WT cells. Considering that *osbpl2b* is an orthologous gene of human *OSBPL2*^27^, *osbpl2b*-KO zebrafish was also constructed (Fig. S2) and used to investigate AMPK activity in inner ear. Meanwhile, AMPK activation was also assessed by examining phosphorylated ACC-Ser79, which was a direct target of activated AMPK and commonly used as an indicator of AMPK activity in cells. The results showed that phosphorylated AMPK-Thr172 and phosphorylated ACC-Ser79 were both detected at lower levels in *Osbpl2*-KO cell (Fig. 2a). A similar effect was observed in *osbpl2b*-KO zebrafish inner ear (Fig. 2b). The protein levels of *Srebp2*, *Hmgcr* and *Hmgcs1*, key regulators in AMPK signaling pathway and cholesterol biosynthesis, were also measured in *Osbpl2/osbpl2b*-KO mutants. The expression of *Srebp2/srebp2*, *Hmgcr/hmgcr* and *Hmgcs1/hmgcs1* in mRNA and protein levels were increased both in *Osbpl2*-KO cells (Fig. 2c, Fig. S3a) and *osbpl2b*-KO zebrafish inner ear (Fig. 2d, Fig. S3b). Besides, the cleavage processing of *Srebp2/srebp2*, as reflected by the amounts of the precursor (126 kDa) and nuclear active forms (55 kDa) were determined. The expression of nuclear *Srebp2/srebp2* were markedly increased both in *Osbpl2*-KO cells (Fig. 2c) and *osbpl2b*-KO zebrafish inner ear (Fig. 2d). In addition, as expected, significant increases of TC content were observed in *Osbpl2*-KO cells (Fig. 2e) and *osbpl2b*-KO zebrafish inner ear (Fig. 2f). The above results indicated that *Osbpl2/osbpl2b*-KO led to the inhibition of AMPK activity and aberrant cholesterol biosynthesis/accumulation in auditory OC1 cells and zebrafish inner ear.

### *Osbpl2*-KO increases cholesterol biosynthesis by suppressing AMPK signaling pathway

**Fig. 3:**
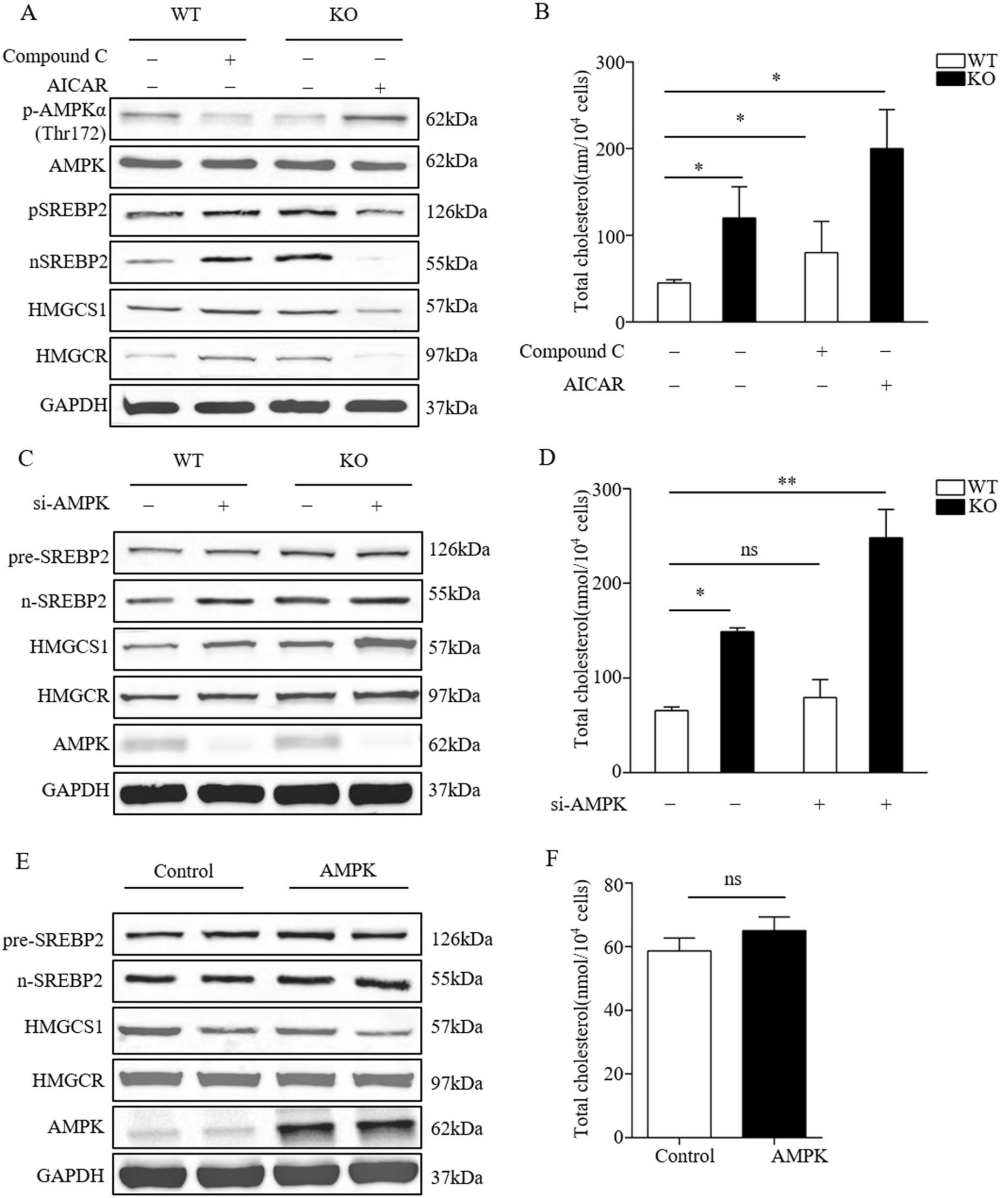
*Osbpl2/osbpl2b*-KO enhanced cholesterol biosynthesis by inhibiting AMPK signaling pathway. **a** Western blot analysis of AMPK (total and Thr172), pre-SREBP2, n-SREBP2, HMGCS1, HMGCR and GAPDH in *Osbpl2*-KO/WT OC1 cells treated with 0.5 μM AICAR (+) or 5 nM Compound C (+) or plain vehicle. **b** Total cholesterol levels in *Osbpl2*-KO/WT OC1 cells treated with 0.5 μM AICAR (+) or 5 nM Compound C (+) or plain vehicle for 24 h (mean ± SEM, *n* = 3), *p < 0.05. **c** Western blot analysis of AMPK, pre-SREBP2, n-SREBP2, HMGCS1, HMGCR and GAPDH in *Osbpl2*-KO/WT OC1 cells treated with AMPK siRNA (+, 100pM) or plain vehicle (−) for 24 h. **d** Total cholesterol levels in *Osbpl2*-KO/WT OC1 cells treated with AMPK siRNA (+, 100pM) or plain vehicle (−) for 24 h (mean ± SEM, *n* = 3), *p < 0.05, **p < 0.01, ns: not significant. **e** Western blot analysis of AMPK, pre-SREBP2, n-SREBP2, HMGCS1, HMGCR, and GAPDH in OC1 cells treated with or without AMPK expression plasmid (5 ng) for 48 h. **f** Total cholesterol levels in OC1 cells treated with or without AMPK expression plasmid (5 ng) for 48 h (mean ± SEM, *n* = 3), ns: not significant

Inhibition of AMPK was conducted in OC1 cells to validate its effect on cholesterol biosynthesis. OC1 cells were treated with 5 nM Compound C, an inhibitor of AMPK, and the levels of phosphorylated AMPK-Thr172, *Srebp2*, *Hmgcr*, and *Hmgcs1* were determined by Western blot analysis. The results showed that the inhibition of AMPK led to a decrease of phosphorylated AMPK-Thr172, but resulted in the up-regulation of nuclear *Srebp2, Hmgcr,* and *Hmgcs1* (Fig. 3a). Meanwhile, a significant increase of intracellular TC content was detected by the treatment with Compound C (Fig. 3b). On the other hand, we wanted to know whether cholesterol synthesis returned to normal levels after AMPK activity inhibition was rescued in *Osbpl2*-KO OC1 cells. The results showed that after AMPK re-activation, the expression of phosphorylated AMPK-Thr172, nuclear *Srebp2*, *Hmgcs1* and *Hmgcr* showed a tendency to return normal, and cholesterol content also recovered (Fig. 3a, b). These results suggested that AMPK might play an important role in cholesterol biosynthesis.

To further confirm the effect of AMPK on cholesterol biosynthesis, AMPK siRNA was used to silence the expression of AMPK. Silencing AMPK up-regulated the expression of nuclear *Srebp2*, *Hmgcr*, and *Hmgcs1* (Fig. 3c), leading to increased cellular TC levels (Fig. 3d). AMPK overexpression had little effect on the expression of nuclear *Srebp2*, *Hmgcr*, and *Hmgcs1* (Fig. 3e) as well as cholesterol biosynthesis (Fig. 3f). These results indicated that the biosynthesis of cholesterol was related to the activity of AMPK but independent of the AMPK expression levels. Therefore, it was suggested that OSBPL2 plays an important role in the regulation on cholesterol biosynthesis through AMPK signaling pathway, and *OSBPL2* deficit induced aberrant cholesterol biosynthesis/accumulation by inhibiting AMPK activity.

### OSBPL2 interacts with ATIC to regulate AMPK activity

**Fig. 4:**
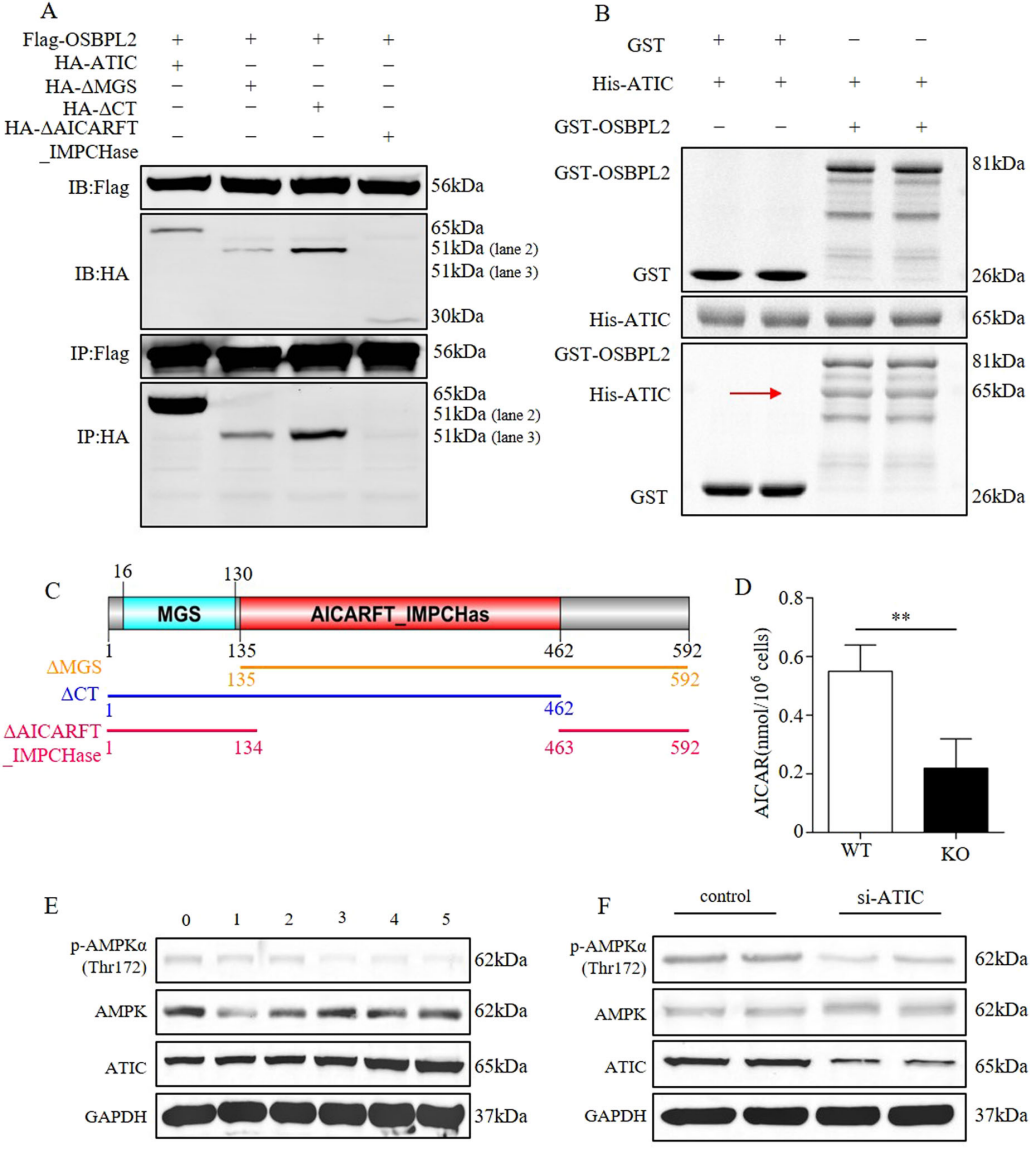
OSBPL2 interacts with ATIC to regulate AMPK activity. **a** Co-IP assay verified the interaction of OSBPL2 with ATIC or truncation (ΔMGS, ΔAICARFT_IMPCHase, ΔCT). HEK293Ta cells expressing HA-ATIC/HA-ATIC-truncations and Flag-OSBPL2 or as negative control. **b** Pulldown assays verifying the interaction of OSBPL2 with ATIC. *E. coli* expressing His-ATIC were lysed and subjected to GST-OSBPL2 pulldown. Plain GST was used as a negative control. **c** Schematic representation of the truncated ATIC proteins. **d** AICAR levels in *Osbpl2*-KO/WT OC1 cells (mean ± SEM, *n* = 3), **p *<* 0.01. **e** Western blot analysis of AMPK (total and Thr172), ATIC and GAPDH in OC1 cells treated with or without ATIC expression plasmid (0, 1, 2, 3, 4, 5 μg) for 48 h. **f** Western blot analysis of AMPK (total and Thr172), ATIC and GAPDH in OC1 cells treated with or without ATIC siRNA (100 pM) for 24 h

The regulatory mechanisms of OSBPL2 on AMPK activity were further investigated by proteomic analysis of the OSBPL2 interactome. Flag-tagged OSBPL2 was expressed in HEK293Ta cells, followed by a pulldown of complexes with anti-Flag magnetic beads. Mass spectrometric analysis identified 47 proteins that were found specifically in the Flag-OSBPL2 complexes (Table S1). Among these binding partners of OSBPL2, ATIC, a bifunctional purine biosynthesis protein, is known to be the key AMPK effector. The interaction of OSBPL2 with ATIC was validated by Co-IP. Abundant HA-ATIC was detected in Flag-OSBPL2 complexes, but absent in the negative control with plain Flag (Fig. 4a), supporting a specific interaction of ATIC with OSBPL2. Similarly, GST pulldown assay showed that GST-OSBPL2 interacted with His-ATIC, but no interactions were detected in the negative control with plain GST (Fig. 4b). The truncated ATIC was used to determine the binding site of ATIC interacting with OSBPL2 (Fig. 4c). The OSBPL2-ATIC interaction remained when ATIC lacked methylglyoxal synthase-like (MGS) domain and carboxyl terminal (CT) domain, but not detected at the absence of 5-aminoimidazole-4-carboxamide ribonucleotide formyltransferase and inosine monophosphate (IMP) cyclohydrolase (AICARFT_IMPCHase) domain (Fig. 4a). These results indicated that OSBPL2 interacted with ATIC and the AICARFT_IMPCHase domain of ATIC was essential for OSBPL2-ATIC interaction.

ATIC is a rate-limiting enzyme and affects intracellular levels of AICAR, which behaves as an AMP analog that allosterically activates AMPK. ATIC is also capable of affecting AMPK activity by regulating the ninth step of de novo purine biosynthesis^28,29^. It was noteworthy that the AICAR level was decreased in *Osbpl2*-KO cells (Fig. 4d). In addition, the level of phosphorylated AMPK-Thr172 decreased in ATIC-overexpressing OC1 cells (Fig. 4e), but increased in ATIC-siRNA knockdown OC1 cells (Fig. 4f). To determine the effect of ATIC on AMPK activity, the AICAR levels were measured in the case of ATIC overexpression or knockdown. The results showed that AICAR was decreased in ATIC-overexpressed OC1 cells (Fig. S4a), while increased in ATIC-siRNA-knockdown OC1 cells (Fig. S4b). These results suggested that the OSBPL2 deficit led to the destruction of the OSBPL2-ATIC interaction, resulting in decreased levels of AICAR and inhibition of AMPK activity.

### Intracellular cholesterol accumulation induces ROS and cause mitochondrial damage

**Fig. 5:**
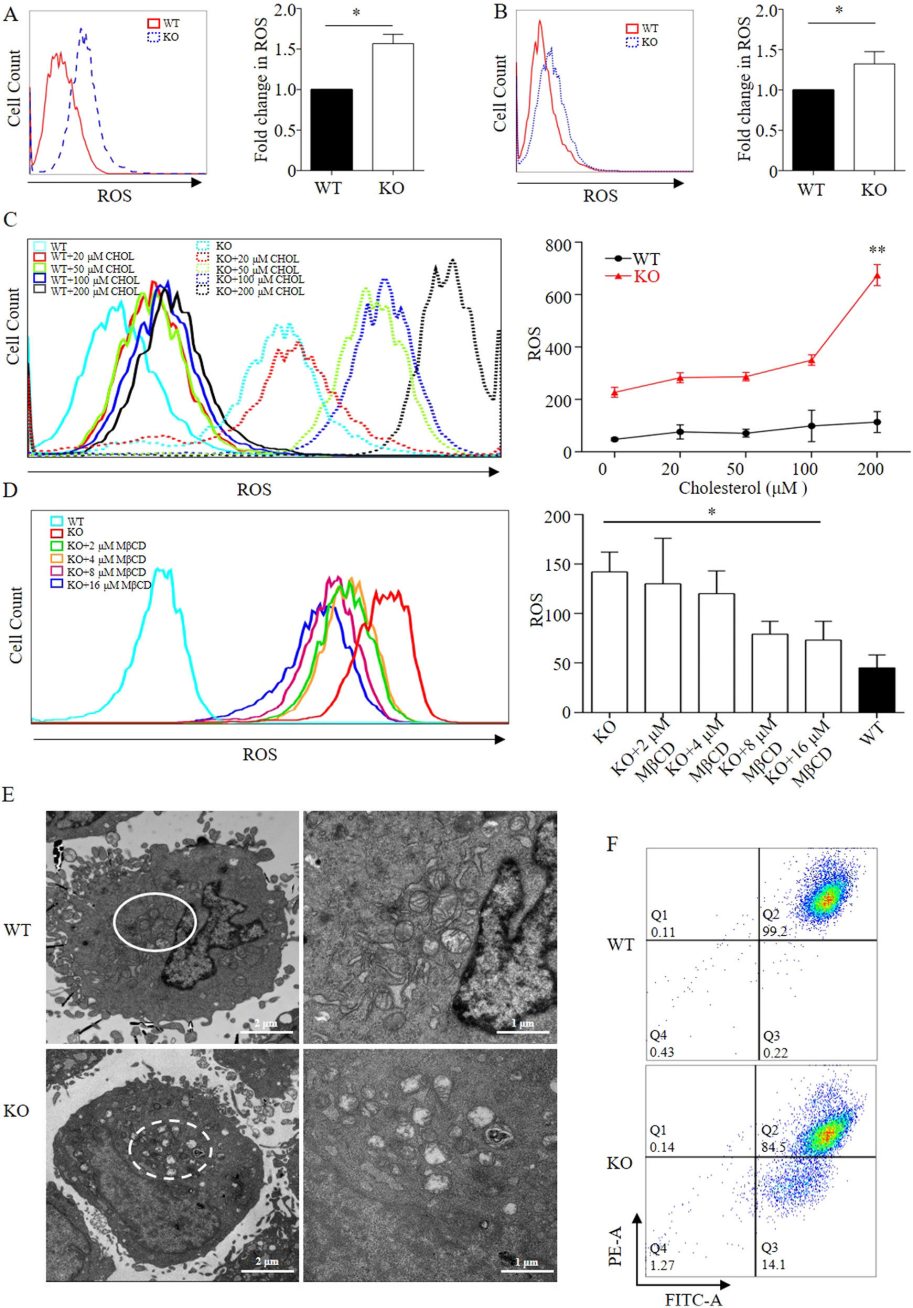
Intracellular cholesterol accumulation induces ROS and causes mitochondrial damage. **a** Mitochondrial ROS levels in *Osbpl2*-KO/WT OC1 cells. Left: representative histogram for CellROX™ Deep Red staining. Right: relative fold changes of ROS (mean ± SEM, *n* = 3), *p < 0.05. **b** Mitochondrial ROS levels in *osbpl2b*-KO/WT zebrafish inner ear. Left: representative histogram for CellROX™ Deep Red staining. Right: relative fold changes of ROS (mean ± SEM, *n* = 3), *p < 0.05. **c** Mitochondrial ROS levels in *Osbpl2*-KO/WT OC1 cells treated with different concentrations of cholesterol (0, 20, 50, 100, 200 μM) for 12 h. Left: representative histogram for CellROX™ Deep Red staining. Right: relative fold changes of ROS (mean ± SEM, *n* = 3), **p < 0.01. **d** Mitochondrial ROS levels in *Osbpl2*-KO/WT OC1 cells treated with different concentrations of MβCD (0, 2, 4, 8, 16 nM) for 12 h. Left: representative histogram for CellROX™ Deep Red staining. Right: relative fold changes of ROS (mean ± SEM, *n* = 3), *p < 0.05. **e** The cellular ultrastructure as analyzed by transmission electron microscopy (TEM) in *Osbpl2*-KO/WT OC1 cells. **f** The membrane potential levels of *Osbpl2*-KO/WT OC1 cells stained by JC-1(10 μM)

Considering the effect of excessive cholesterol on intracellular ROS production, the increased TC content induced by *Osbpl2/osbpl2b*-KO might also be of sufficient magnitude to affect ROS levels. As was expected, the basal ROS levels of *Osbpl2*-KO OC1 cells were ~50% higher than that of WT controls (Fig. 5a). Similarly, *osbpl2b*-KO increased the ROS levels by 30% in zebrafish inner ear (Fig. 5b). A significant increase of ROS levels was also detected in *Osbpl2*-KO OC1 cells treated with different concentrations of cholesterol (Fig. 5c). However, the ROS levels in *Osbpl2-KO* OC1 cells treated with MβCD showed a tendency to return normal. (Fig. 5d). The effect of ROS on the subcellular features was assessed in *Osbpl2*-KO cells. Compared with WT controls, swollen mitochondria with missing cristae were observed in *Osbpl2-*KO cells (Fig. 5e). In addition, the mitochondrial membrane potential was also detected decreased in *Osbpl2*-KO OC1 cells (Fig. 5f). Meanwhile, several antioxidant genes, including *Sod1*, *Sod2*, and *Cat*, were significantly downregulated in *Osbpl2*-KO OC1 cells (Fig. S5). These results strongly suggested the mitochondria damage in *Osbpl2*-KO OC1 cells.

**Fig. 6:**
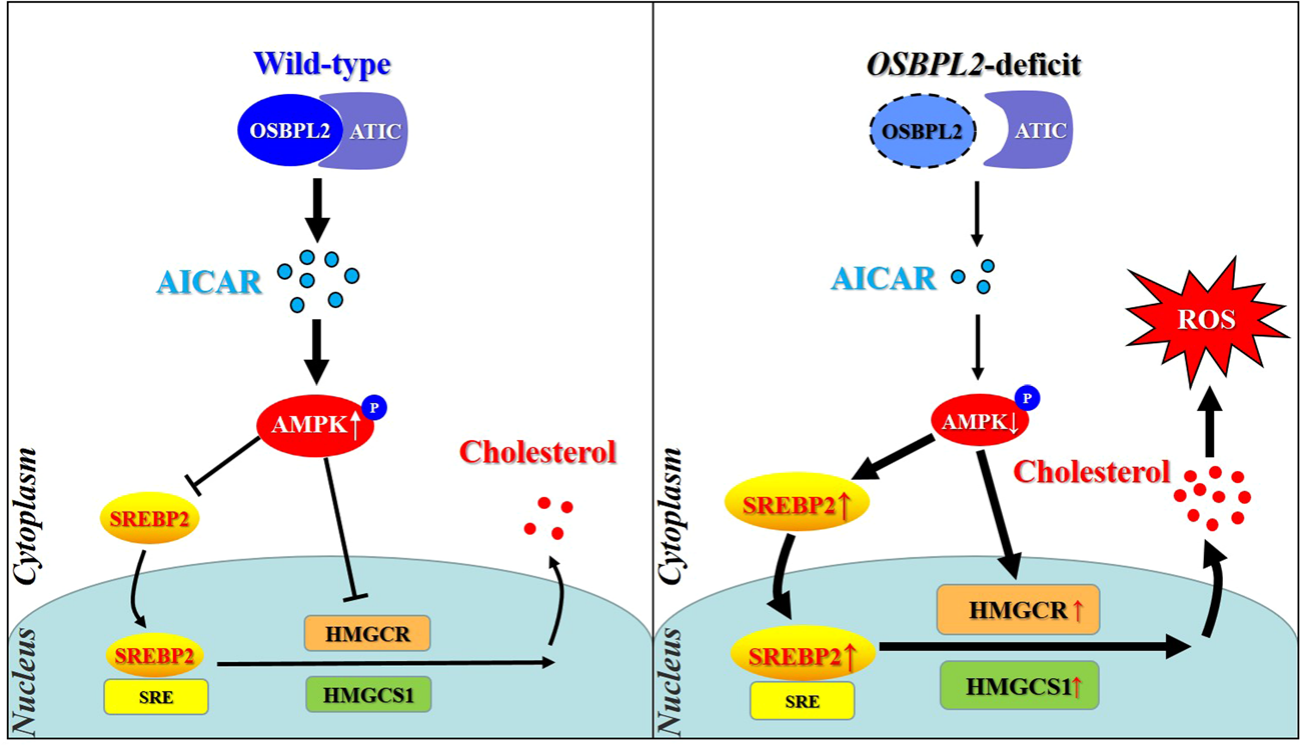
Proposed regulatory mechanism of OSBPL2 on intracellular cholesterol biosynthesis and ROS production. SRE: sterol response element. ROS: reactive oxygen species

The above results suggested that the OSBPL2-ATIC interaction contributed to intracellular AICAR accumulation in WT cells, which activated AMPK signaling pathway and controlled the nuclear transfer of SREBP2 to bind sterol response element (SRE)^30,31^, resulting in regular cholesterol biosynthesis. In addition, AMPK regulated cholesterol synthesis by directly controlling the activity of HMGCR^32^. On the other hand, OSBPL2 deficit interfered the OSBPL2-ATIC interaction and reduced the intracellular AICAR level as well as AMPK activity, which enhanced nuclear transfer of SREBP2 and upregulated the expression HMGCR and HMGCS1, resulting in excessive cholesterol biosynthesis and ROS accumulation (Fig. 6).

## Discussion

Since *OSBPL2* was identified as the ADNSHL-causative gene^21,22^, it has drawn much attention to the molecular function of OSBPL2 implicated in multiple biological processes. The current studies revealed the role of OSBPL2 in lipid/cholesterol transport and metabolism as well as cell signaling in diverse cell types, which suggest that OSBPL2 might play an important role in the regulation of intracellular cholesterol-homeostasis. To gain a deep understanding of the physiologic function of OSBPL2 in inner ear, we employed *Osbpl2*-KO/knockdown OC1 auditory cells and *osbpl2b*-KO zebrafish to investigate the regulatory mechanism of OSBPL2 in cholesterol-homeostasis. The RNA-seq analysis revealed the alteration of *Osbpl2*-KO OC1 cells in the biological process categories: lipid transport, lipid metabolic process, lipid storage and cholesterol transport, corresponding to estradiol and cholesterol biosynthesis. KEGG Pathway analysis showed that AMPK signaling pathway was the most significant pathway. *Osbpl2*-KO resulted in up-regulation of *Srebp2*, *Hmgcr* and *Hmgcs1*, key genes in the sterol biosynthetic pathway and associated with AMPK signaling. These results strongly suggested that *Osbpl2/osbpl2b* deficits inhibited AMPK activity and enhanced cholesterol biosynthesis.

AMPK is the downstream component of a kinase cascade that acts as a gauge of cellular energy levels. Recently, accumulating evidence has demonstrated that AMPK is also the key regulator of metabolism, protein synthesis, cell growth and apoptosis, cell polarity and ion flux^33–35^. There was a strong correlation between low AMPK activation status with insulin resistance, obesity and sedentary lifestyle^36–38^. Decreased AMPK activation is implicated in human metabolic disorders associated with increased cancer risk^39^. The previous studies had revealed that the AMPK signaling pathway was closely related to hearing loss. LKB1, upstream kinase of AMPK, is expressed from the cuticular plate to the nuclei of hair cells and contributes to maintaining the development and structural stability of cochlear hair cells and stereocilia bundles^40^. Hill et al. found that long-term phosphorylation of AMPK caused by noise exposure could inversely lead to a decreased auditory function along with the loss of hair cell and synaptic ribbon^41^. In fact, AMPK controls cellular lipid metabolism through direct phosphorylation of ACC and ACC1. AMPK also phosphorylates and inhibits SREBP1/2 and HMGCR, which leads to a preprogramming of lipid and intracellular sterol biosynthesis^32,42^. In the present work, AMPK inhibition increased intracellular cholesterol synthesis, which was observed both in *Osbpl2/osbpl2b*-KO mutants. It was suggested that inhibition of AMPK promotes nuclear transfer of SREBP2, which promotes the expression of *Hmgcs1* and *Hmgcr*.

The interaction of OSBPL2 with ATIC, the key effector of AMPK activity, further confirmed the regulatory role of OSBPL2 on AMPK activity. We found that OSBPL2 combined with the AICARFT_IMPCHas domain of ATIC and OSBPL2-deficient resulted in the inhibition of AMPK activity. ATIC is a cytosolic enzyme (64 kDa) containing a transformylase domain (residues 200–593), which transfers a formyl group to the AMP analog AICAR to produce the intermediate formyl-AICAR (FAICAR) and IMP^43,44^. AICAR is a nucleoside that is converted inside the cells to its mono-phosphorylated form, 5-amino-4-imidazolecarboxamide ribotide (ZMP) by adenylate kinase. Thus, AICAR behaves as an AMP analog that allosterically activates AMPK. Recent studies demonstrated that inhibition of ATIC led to a subsequent rise in intracellular ZMP and resulted in the increased AMPK activity^45^. Furthermore, Li et al. found that ATIC overexpression inhibited AMPK activity, and homodimerization of ATIC was essential for the AICAR transformylase (AICART) activity, as the active site of this enzyme was formed at the interface of two interacting ATIC monomers with each molecule contributing residues^29^. Daniel et al. found that an inhibitor of ATIC homodimerization up-regulated intracellular ZMP via a metabolic block on the ninth step of the de novo purine biosynthesis pathway^28^. In the present work, we found that OSBPL2 binds to the AICARF domain of ATIC and regulates the cellular levels of AICAR. The present data were consistent with a model in which OSBPL2 deficits led to the exposure of the AICARF domain, inhibited the cellular levels of AICAR, and suppressed AMPK activity.

Beyond the role of OSBPL2 in lipid/cholesterol metabolism and transport, the present study uncovered *OSBPL2* as a key regulator in cellular cholesterol-biosynthesis using auditory OC1 cells and zebrafish models. Consistent with RNA-seq analysis, we found that the OSBPL2 deficits led to an inhibition of AMPK activity and aberrant cellular cholesterol biosynthesis, which suggested the deregulation of cholesterol homeostasis. Increasing evidences had suggested the importance of lipid/cholesterol-homeostasis in auditory function. Some genetic syndromes, such as Niemann-Pick type C and Smith-Lemli-Opitz, that affect cholesterol intracellular transport/synthesis display devastating neurological phenotypes including SNHL^46,47^. The epidemiology studies had suggested the potential association of hypercholesterolemia and SNHL. Interestingly, it was found that Apolipoprotein E (ApoE) knockout mice developed remarkable hyperlipedimia, atherosclerosis and hearing impairment, which might share the similar pathogenesis of OSBPL2 deficit in SNHL^48^. In our study, excessive cholesterol levels and increased ROS were both detected in *Osbpl2*-KO OC1 cells and *osbpl2b*-KO zebrafish inner ear. ROS are endogenous products of metabolism and essential as the second message in cellular signaling involved in multiple cellular process. However, excessive ROS accumulation can damage cellular proteins, lipids and DNA, leading to cell dysfunction, senescence and even cell death^49–51^. Gonzalo Alba et al reported that excessive cholesterol enhanced ROS production in human neutrophils^52^. In the hyperlipidemia/hyperlipedimia mice/rats models, it was found that long-term high-fat diets led to the activation/elevation of NADPH Oxidase Catalytic Subunit-Like 3 (NOX3), uncoupling protein 2 (UCP2) and uncoupling protein 3 (UCP3), causing mitochondrial damage and apoptosis in inner ear^53,54^. Mitochondria are a major source of ROS, but easily damaged by ROS. This mitochondrial oxidative damage contributes to cellular dysfunction or cell death in various diseases^55^. Our study suggested that *Osbpl2/osbpl2b-KO* enhanced the ROS production and led to mitochondrial damage and membrane potential reduction in auditory OC1 cells, which might be implicated in the pathogenesis of OSBPL2 deficit in SNHL development.

In summary, the present work uncovered the regulatory mechanism of OSBPL2 on cholesterol-biosynthesis/homeostasis in OC1 auditory cells and inner ear of zebrafish. It was suggested that OSBPL2 and other key regulators/effectors in AMPK signaling pathway were critical for cholesterol-homeostasis in inner ear and maintenance of hearing function. Our findings contributed to elucidating the pathogenesis/physiopathology of *OSBPL2* mutation and the potential linkage of deregulation of cholesterol-homeostasis and SNHL.

## Materials and methods

### Antibodies and reagents

Antibodies for adenosine 5′-monophosphate (AMP)-activated protein kinase (AMPK/Ampk) (#2793), Flag-Tag (#2368), His-Tag (#12698), GST-Tag (#2624), GAPDH (#5174) and phosphospecific antibodies for AMPK/Ampk -pThr172 (#2535) were purchased from Cell Signaling Technology (Danvers, MA, USA). Antibodies for HMGCR/Hmgcr (#ab174830), 5-aminoimidazole-4-carboxamide ribonucleotide formyltransferase/inosine monophosphate cyclohydrolase (ATIC) (#ab188321), HA-Tag (#ab1424) and phosphospecific antibodies for acetyl-CoA carboxylase (ACC/Acc) pSer79 (#ab68191) were purchased from Abcam (Cambridge, UK). Antibodies for ACC/Acc (#21923-1-AP), 3-hydroxy-3-methylglutaryl-CoA synthase 1 (HMGCS1) (#17643-1-AP) and sterol regulatory element-binding protein 2 SREBP2/Srebp2 (#A13049) were from Proteintech (Chicago, IL, USA). Antibody for Gapdh (#GTX100118) was purchased from GeneTex (Alton, CA, USA). IRDye 800CW goat anti-Mouse IgG (#926-32213) and IRDye 680LT goat anti-Rabbit IgG (#926-68021) were purchased from LI-COR Biosciences (Lincoln, Nebraska, USA). Cholesterol (#C8667) and methyl-β-cyclodextrin (#C4555) were purchased from Sigma-ldrich (St. Louis, MO, USA). Aminoimidazole carboxamide ribonucleotide (AICAR) (#S1802) and Compound C (#S7840) were purchased from Selleckchem (Houston, TX, USA).

### Animals

*Osbpl2b*-KO and wild-type (WT) zebrafish were maintained using standard procedures and used in accordance with the protocols approved by the Institutional Animal Care and Use Committee (IACUC) of the Nanjing Medical University. The fish were kept at 28 °C in buffered reverse osmosis water with a standard light/dark cycle of 14 h/10 h and fed Aquatox Fish Diet flakes (Zeigler, China) twice per day, supplemented with brine shrimp.

### Cell culture and transfection

*Osbpl2b*-KO/WT OC1 cells were grown in Dulbecco’s Modified Eagle’s Medium (DMEM, Gibco, USA) supplemented with 10% Foetal Bovine Serum (FBS, Gibco, USA) at 33 °C with 10% CO_2_, subcultured every 2 day at a density of 1/4 or every 3 d at a density of 1/8 and discarded after passage 8. HEK293Ta cells were cultured in the above medium at 37 °C with 5% CO_2_. Plasmids were transfected into cells using Lipofectamine 3000 Reagent (Invitrogen, USA) when the cells achieved 70%–90% confluent. SiRNAs were transfected into cells using X-tremeGENE siRNA Transfection Reagent (Roche, USA) at 30–50% of cell confluent.

### Quantitative real-time polymerase chain reaction

Cells or tissues were homogenized in TRIzol Reagent (Life, USA) and total RNA were extracted according to the manufacturer’s instructions. Complementary DNA (cDNA) was synthesized via HiScript II One Step RT-PCR Kit (Vazyme, China). The quantitative real-time polymerase chain reaction (qRT-PCR) was performed on a StepOne Plus system (Applied Biosystems, USA) using ChamQ SYBR qPCR Master Mix (Vazyme, China). The comparative CT method (2^−ΔΔCT^) was used to analyze gene expression. GAPDH was used as an internal control. The primer sequences were listed in Supplemental Table S2.

### Western blot analysis

Cells were washed three times with cold PBS and then lysed with RIPA Lysis Buffer (Beyotime, China). Lysates were sonicated for 10–15 s to shear DNA, heated to 100 °C for 10 min and centrifuged for 10 min at 13,000*×g*. The protein content in the supernatant were quantified using BCA Protein Assay Kit (Beyotime, China). For protein separation, samples were prepared by mixing 5xSDS loading buffer with 10–25 μg of cell lysates and separated using SDS/PAGE 10–15% gels. Proteins were transferred to PVDF membranes, blocked with TBS-T containing 5% milk powder for 2 h and then incubated with primary antibody overnight at 4 °C. The post-transferred Membranes were washed for three times, followed by labeling of secondary antibodies. Signals were visualized with Odyssey® CLx Imaging System (LI-COR, USA). Due to difficulties of stripping, some plots were blotted with two different membranes but from same cell lysates.

### siRNA knockdown

Cells were grown for 12 h to obtain 30–50% confluence. The siRNA and X-tremeGENE siRNA Transfection Reagent (Roche, USA) were separately mixed with 250 μl of Opti-MEM ® I Reduced Serum Medium (Gibco, USA) for 5 min. The two mixtures were added to the cells and incubated at 37 °C for 6 h. Then, the serum-free medium was changed to 10% serum medium for further study. All siRNA synthesized by RiboBio (Guangzhou, China).

### RNA sequencing and data analysis

Cells were lysed and total RNA was extracted as mentioned above. Samples were then submitted to Biomarker Technologies (Beijing, China), where mRNA enrichment and library preparation were performed. Samples were barcoded and run on lllumina HiSEq. Reads from RNA sequencing (RNA-seq) were mapped to Rattus norvegicus genome Rnor_6.0 (ftp://ftp.ensembl.org/pub/release-95/fasta/rattus_norvegicus/) by using STAR (v2.5.2a) with 2% maximum mismatch. Fragments per kilobase of transcript per million fragments mapped (FPKM) and differentially expressed genes (DEGs) (*q*-value < 0.05) were generated by Cuffdiff (v2.2.1) with default parameters. The genes sensitive to *Osbpl2* deficit were filtered by a minimum fold change (×2) and a maximum false discovery rate (×0.01) and subjected to Visualization and Integrated Discovery (DAVID) v6.8 for KEGG pathway analysis and Gene ontology (GO) analysis.

### Co-immunoprecipitation (Co-IP)

HEK293Ta cells expressing the constructs of HA-ATIC and Flag-OSBPL2 were washed three times with cold PBS and then lysed with RIPA Lysis Buffer (Beyotime, China) containing complete protease inhibitors (Beyotime, China) and PhosStop (Beyotime, China) for 30 min. Protein contents in the supernatant were incubated with Anti-FLAG® M2 Magnetic Beads (Sigma-Aldrich, Germany) overnight at 4 °C. The samples were washed using RIPA Lysis Buffer (Beyotime, China) three times. Immobilized protein complexes were eluted by denaturation in 5x SDS loading buffer at 100 °C for 10 min and then assayed by Western blot. The protein bands were retrieved and subjected to LC-MS/MS analysis (PTM Biolabs Inc., Hanzhou, China).

### GST pulldown

The proteins of His-ATIC and GST-OSBPL2 were synthesized by Zoonbio Biotechnology (Nanjing, China). Protein samples were incubated with Anti-His, Anti-GST and Protein A/G overnight at 4 °C and washed with RIPA Lysis Buffer (Beyotime, China) three times. Immobilized protein complexes were eluted and assayed by Co-IP.

### AICAR measurement

AICAR was measured according to the manufacturer’s instructions of AICAR ELISA Kit (Elabscience, China). Briefly, OC1 cells were washed with pre-cooled PBS and dissociated by trypsin. The cell suspension was collected, centrifuged at 1000 × *g* for 5 min, and suspended using pre-cooled PBS. The cells were fully lysed by repeated freeze-thaw processes. Then the samples were added to the 96-well and incubated at 37 °C for 90 min. The supernatant was removed, and the remained solution was added with 100 μL Biotinylated Detection Ab. The solution was aspirated or poured from each well and 350 μL of wash buffer was added to each well. The samples were added with 100 μL HRP conjugate, incubated 30 min at 37 °C and washed 3 × 5 min by PBS. Then the samples were added with 90 μL Substrate Reagent, incubated for 15 min at 37 °C and treated with 50 μL of Stop Solution. The optical density (absorbance at 450 nm) of the samples were determined using a micro-plate reader.

### Total cholesterol assay

The cells (1 × 10^6^ cells) and tissues (0.1 g) were added to 1 ml of isopropanol for homogenization in an ice bath and centrifuged at 8000 × *g* for 10 min at 4 °C. The supernatant was used as the total cholesterol (TC) test solution. The reaction solution (50 μL of TC standard or TC test solution) and TC working solution (150 μL), were mixed in a 1 mL glass cuvette, kept for 24 h and then used to determine the absorbance (A) at 500 nm. TC content in tissues: TC (μmol/g) = 0.05 × *A*_samples_/*A*_standard_. TC content in cells: TC (μmol/10^4^ cell) = 0.005 × *A*_samples_/*A*_standard_.

### ROS measurements

The test cells or tissues were treated with or without drug and cultured for the recommended time. The cultured cells or tissues were then incubated with DCFDA (Beyotime, China) or CellROX™ Deep Red Reagent (ThermoFisher, USA) at a final concentration of 5 μM, followed by analysis of flow cytometry. The ROS levels were quantified as the mean florescence intensity (MFI). All flow cytometry was conducted using BD FACSCalibur (Becton, Dickinson and Company, USA) and analyzed with FlowJo software (v10.5.3).

### Transmission electron microscopy assay

For transmission electron microscopy (TEM) study, cells were fixed in 2% glutaraldehyde, and subjected to post-fixation with 1% osmium tetroxide in 0.1 M cacodylate. The samples were embedded overnight with a mixture solution of 1/1 ratio of propylene oxide/epoxy resin, replaced with 100% epoxy resin and then polymerized in a dry oven at 60 °C. Ultrathin sections (70 nm thick) from the embedded samples were imaged by TEM (JEOL-101, Japan).

### Mitochondrial membrane potential assay

JC-1 probe (Beyotime, China) was employed to measure mitochondrial depolarization in OC1 cells. Cells cultured in six-well plates for 12 h, then incubated with JC-1 staining solution (5 μg/ml) at 37 °C for 30 min. Cells were washed three times with cold PBS. Mitochondrial membrane potential was measured by flow cytometry. Mitochondrial depolarization was indicated by an increase of the green/red fluorescence intensity ratio.

### Statistical analysis

All data were presented as the mean ± standard error of the mean (SEM). One-way ANOVA and Bonferroni’s post-tests were performed to compare the differences between the groups. Other statistical significance was determined using between-group *t*-test. A *p*-values < 0.05 was regarded as statistically significant. Statistical analysis was performed using GraphPad Prism v7 (San Diego, CA, USA).

## Supplementary information

Supplementary materials.
