# Supplementary material for: Deletion of OSBPL2 in auditory cells increases cholesterol biosynthesis and drives reactive oxygen species production by inhibiting AMPK activity
Source: Cell Death Dis. 2019 Aug 19;10(9):627. doi: 10.1038/s41419-019-1858-9 (PMC6700064; doi:10.1038/s41419-019-1858-9)
Supplement: Supplementary file 1 — Supplementary materials. [file 41419_2019_1858_MOESM1_ESM.docx]

**Supplementary Figure Legends:**

**Fig. S1 Volcano plots of significant DEGs in the KO/WT samples**. Red and blue dots indicated upregulation and downregulation of genes.

**Fig. S2 Generation of *osbpl2b* knockout zebrafish.** (A) Schematic diagram of *osbpl2b* gene, the CRISPR/CAS9 gRNA sequence and PAM domain. (B) Representative sequencing showing modifications at the CRISPR/CAS9 cutting site on exon 6 of the *osbpl2* gene (C) The predicted protein products of *osbpl2* in the mutants and their wild-type. (D) Western blot revealed *osbpl2* expression in *osbpl2*-KO/WT zebrafish inner ear tissues.

**Fig. S3 *Osbpl2/osbpl2b*-KO increased the expression of key genes for cholesterol synthesis.** (A) mRNA expression of *Srebf2*, *Hmgcr* and *Hmgcs1* in *Osbpl2*-KO/WT OC1 cells. (B) mRNA expression of *srebf2*, *hmgcr* and *hmgcs1* in *osbpl2b*-KO/WT OC1 cells. mRNA expression was normalized to *Gapdh*/gapdh (mean ± SEM, n=3), *p < 0.05.

**Fig. S4 ATIC regulated the AICAR levels in OC1 cells.** (A) The AICAR levels in OC1 cells, which were overexpressed with different amounts of ATIC plasmid (n=3), *p < 0.05. (B) The AICAR levels in OC1 cells with ATIC-knockdown by siRNA (n=3), *p < 0.05.

**Fig. S5 mRNA expression of *Sod1*, *Sod2*, *Gpx1 and* *Cat* in *Osbpl2*-KO/WT OC1 cells.** mRNA expression normalized to Gapdh (mean ± SEM, n=3), *p < 0.05, ns - not significant.


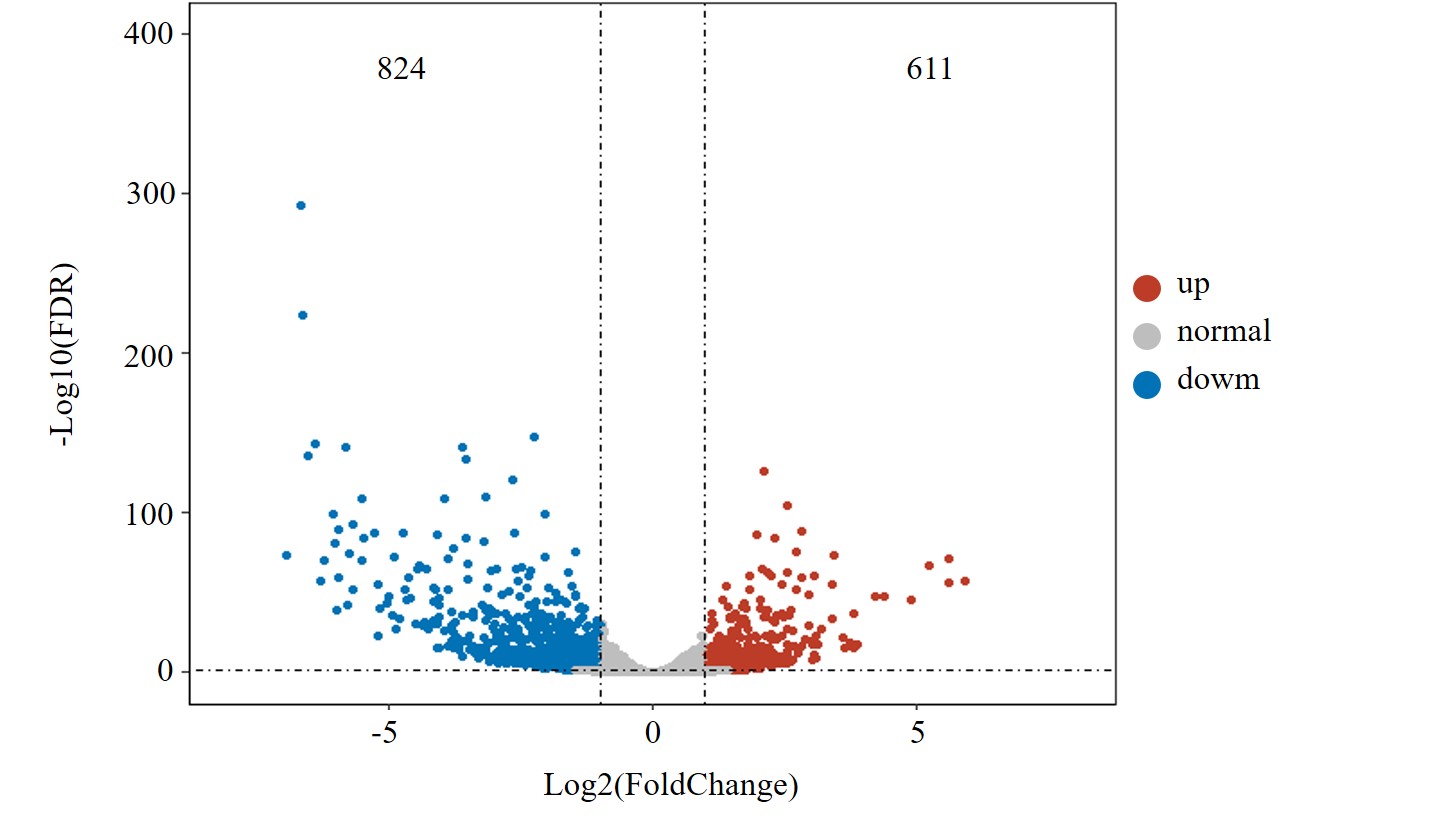


Fig. S1


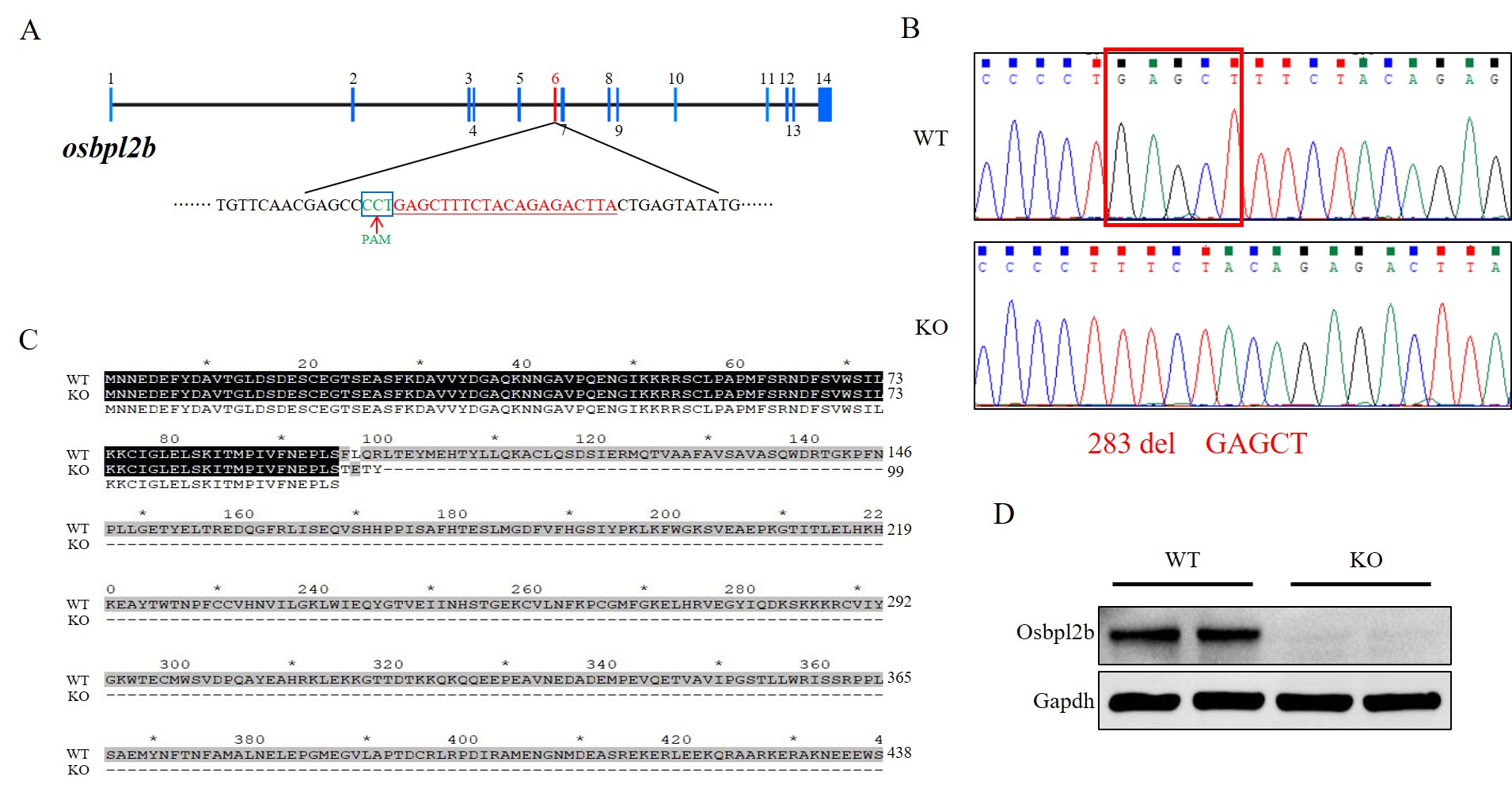


Fig. S2


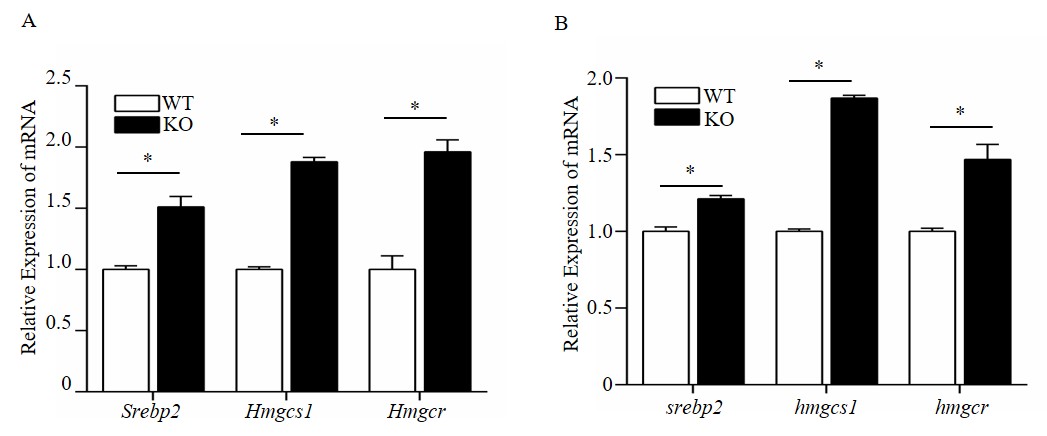


Fig. S3


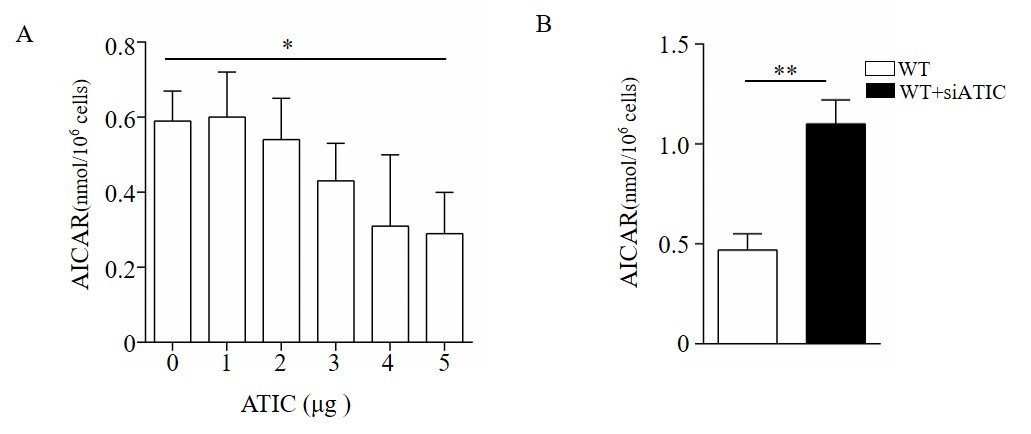


Fig. S4


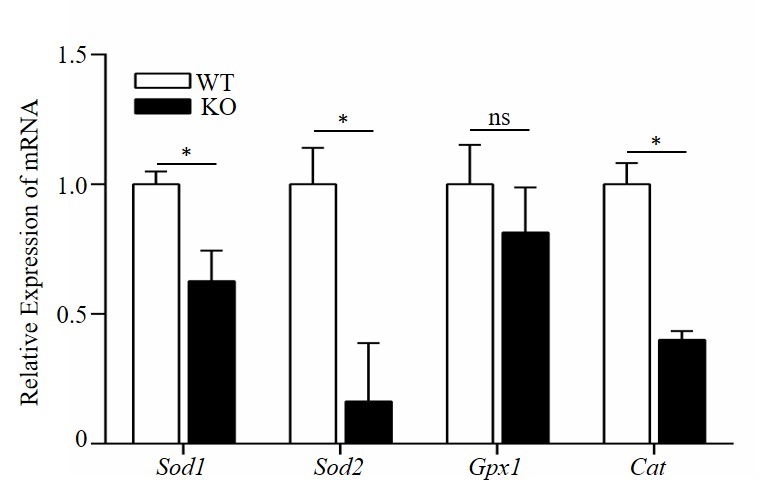


Fig. S5

**Supplementary Table Captions**

Table S1 Proteomic Analysis of proteins interacting with OSBPL2

Table S2 qPCR Primers Sequences

Table S1 Proteomic Analysis of proteins interacting with OSBPL2

| **Accession** | **Description** | **MW [kDa]** | **Score** | **Coverage** | **# Unique Peptides** | **# Peptides** | **# PSMs** | **# AAs** | **calc. pI** |
| --- | --- | --- | --- | --- | --- | --- | --- | --- | --- |
| Q9P0L0 | VAPA | 27.9 | 275.33 | 17.67 | 3 | 4 | 6 | 249 | 8.62 |
| Q9Y3F4 | STRAP | 38.4 | 226.50 | 18.86 | 4 | 4 | 4 | 350 | 5.12 |
| P31939 | ATIC | 64.6 | 210.78 | 9.97 | 5 | 5 | 5 | 592 | 6.71 |
| P56192 | SYMC | 101.1 | 155.85 | 4.22 | 3 | 3 | 3 | 900 | 6.16 |
| P54136 | SYRC | 75.3 | 140.33 | 6.52 | 4 | 4 | 4 | 660 | 6.68 |
| P17812 | PYRG1 | 66.6 | 116.06 | 4.74 | 2 | 2 | 2 | 591 | 6.46 |
| O14980 | XPO1 | 123.3 | 103.02 | 2.24 | 2 | 2 | 2 | 1071 | 6.06 |
| Q14166 | TTL2 | 74.4 | 100.63 | 5.59 | 2 | 2 | 2 | 644 | 5.53 |
| Q13283 | G3BP1 | 52.1 | 98.09 | 3.86 | 1 | 1 | 1 | 466 | 5.52 |
| P46459 | NSF | 82.5 | 92.03 | 4.30 | 3 | 3 | 3 | 744 | 6.95 |
| Q96P70 | IPO9 | 115.9 | 90.91 | 3.55 | 2 | 2 | 2 | 1041 | 4.81 |
| Q9UBB4 | ATX10 | 53.5 | 83.02 | 4.84 | 2 | 2 | 2 | 475 | 5.25 |
| P53621 | COPA | 138.3 | 78.61 | 1.80 | 2 | 2 | 2 | 1224 | 7.66 |
| P46379 | BAG6 | 119.3 | 75.86 | 1.86 | 2 | 2 | 2 | 1132 | 5.60 |
| Q9UHI6 | DDX20 | 92.2 | 75.39 | 1.70 | 1 | 1 | 1 | 824 | 6.95 |
| Q8TEQ6 | GEMI5 | 168.5 | 69.32 | 2.12 | 2 | 2 | 2 | 1508 | 6.62 |
| P33993 | MCM7 | 81.3 | 64.10 | 4.31 | 2 | 2 | 2 | 719 | 6.46 |
| O94905 | ERLN2 | 37.8 | 63.84 | 7.96 | 2 | 2 | 2 | 339 | 5.62 |
| Q13155 | AIMP2 | 35.3 | 61.63 | 6.56 | 2 | 2 | 2 | 320 | 8.22 |
| Q7Z2W4 | ZCCHV | 101.4 | 59.19 | 1.66 | 1 | 1 | 1 | 902 | 8.40 |
| O95373 | IPO7 | 119.4 | 57.52 | 2.02 | 2 | 2 | 2 | 1038 | 4.82 |
| Q13148 | TADBP | 44.7 | 56.05 | 4.35 | 1 | 1 | 1 | 414 | 6.19 |
| P54578 | UBP14 | 56.0 | 54.86 | 2.63 | 1 | 1 | 1 | 494 | 5.30 |
| P48444 | COPD | 57.2 | 51.39 | 2.15 | 1 | 1 | 1 | 511 | 6.21 |
| P45880 | VDAC2 | 31.5 | 50.58 | 6.80 | 1 | 1 | 1 | 294 | 7.56 |
| Q8N684 | CPSF7 | 52.0 | 49.57 | 2.76 | 1 | 1 | 1 | 471 | 8.00 |
| O00139 | KIF2A | 79.9 | 49.28 | 1.56 | 1 | 1 | 1 | 706 | 6.68 |
| O00507 | USP9Y | 290.9 | 48.66 | 0.39 | 1 | 1 | 1 | 2555 | 5.86 |
| Q96CS3 | FAF2 | 52.6 | 47.33 | 2.70 | 1 | 1 | 1 | 445 | 5.62 |
| O60884 | DNJA2 | 45.7 | 46.88 | 2.43 | 1 | 1 | 1 | 412 | 6.48 |
| P62333 | PRS10 | 44.1 | 44.78 | 3.60 | 1 | 1 | 1 | 389 | 7.49 |
| O95831 | AIFM1 | 66.9 | 44.76 | 1.31 | 1 | 1 | 1 | 613 | 8.95 |
| Q15436 | SC23A | 86.1 | 44.67 | 1.57 | 1 | 1 | 1 | 765 | 7.08 |
| Q14004 | CDK13 | 164.8 | 42.92 | 1.06 | 1 | 1 | 1 | 1512 | 9.69 |
| P53618 | COPB | 107.1 | 41.94 | 1.47 | 1 | 1 | 1 | 953 | 6.05 |
| O95159 | ZFPL1 | 34.1 | 40.03 | 2.58 | 1 | 1 | 1 | 310 | 8.07 |
| P05141 | ADT2 | 32.8 | 38.76 | 4.03 | 1 | 1 | 1 | 298 | 9.69 |
| O94915 | FRYL | 339.4 | 38.35 | 0.30 | 1 | 1 | 1 | 3013 | 5.58 |
| Q9BQ67 | GRWD1 | 49.4 | 37.63 | 2.24 | 1 | 1 | 2 | 446 | 4.92 |
| P50402 | EMD | 29.0 | 36.85 | 4.72 | 1 | 1 | 1 | 254 | 5.50 |
| Q8N1I0 | DOCK4 | 225.1 | 36.13 | 0.61 | 1 | 1 | 1 | 1966 | 7.65 |
| Q13409 | DC1I2 | 71.4 | 34.41 | 2.19 | 1 | 1 | 1 | 638 | 5.20 |
| O15027 | SC16A | 233.4 | 34.25 | 0.50 | 1 | 1 | 1 | 2179 | 5.63 |
| Q9NTJ3 | SMC4 | 147.1 | 33.45 | 0.47 | 1 | 1 | 1 | 1288 | 6.79 |
| Q7L1Q6 | BZW1 | 48.0 | 32.56 | 1.43 | 1 | 1 | 1 | 419 | 5.92 |
| Q9BSJ8 | ESYT1 | 122.8 | 29.54 | 0.63 | 1 | 1 | 1 | 1104 | 5.83 |
| P02765 | FETUA | 39.3 | 25.40 | 3.54 | 1 | 1 | 1 | 367 | 5.72 |

Table S2 qPCR Primers Sequence

| **Sequence Name** | **Sequence** |
| --- | --- |
| zebrafish-*srebp2*-F | CAATCAAAGCTCAACGTCAGTG |
| zebrafish-*srebp2*-R | TCATCTGAACAGCCTTACACAG |
| zebrafish-*hmgcs1*-F | AGTTAGCAGGACAGAGAATTGG |
| zebrafish-*hmgcs1*-R | GAGACCAACTTATCCAGAGCAG |
| zebrafish-*hmgcra*-F | ACTTTAATGGAGGCTTCAGGAG |
| zebrafish-*hmgcra*-R | GCACCCAGAACACCTAACATC |
| zebrafish-*gapdh*-F | TGGTCACATGGTATGACAATGA |
| zebrafish-*gapdh*-R | GAGAATGGTCGCGTATCAAAAA |
| rat-*Srebf2*-F | CTATTCCATTGACTCTGAGCCG |
| rat-*Srebf2*-R | CAGGAAGGTGAGGACACATAAG |
| rat-*Hmgcs1*-F | ACTGGTGCAGAAATCTCTAGC |
| rat-*Hmgcs1*-R | TGCCTTTTCCACATCTCTGTC |
| rat-*Hmgcr*-F | CTATTGCACCGACAAGAAACC |
| rat-*Hmgcr*-R | TTACGTCAACCATAGCTTCCG |
| rat-*Cat*-F | CAAGCTGGTTAATGCGAATGG |
| rat-*Cat*-R | TTGAAAAGATCTCGGAGGCC |
| rat-*Cytc*-F | CCCTAAGAGTCTGATCCTTTGTG |
| rat-*Cytc*-R | TCCAGTCTTATGCTTGCCTC |
| rat-*Sod1*-F | TGTGTCCATTGAAGATCGTGTG |
| rat-*Sod1*-R | CTTCCAGCATTTCCAGTCTTTG |
| rat-*Sod2*-F | GGACAAACCTGAGCCCTAAG |
| rat-*Sod2*-R | CAAAAGACCCAAAGTCACGC |
| rat-*Gpx1*-F | GAACCCGATATAGAAGCCCTG |
| rat-*Gpx1*-R | CCATCACCAAGCCAATACCAG |
| rat-Gapdh-F | TCCAGTATGACTCTACCCACG |
| rat-Gapdh-R | CACGACATACTCAGCACCAG |
